# Association between CYP17 T-34C rs743572 and breast cancer risk

**DOI:** 10.18632/oncotarget.23688

**Published:** 2017-12-26

**Authors:** Jing Sun, Hong Zhang, Meiyan Gao, Zhishu Tang, Dongyan Guo, Xiaofei Zhang, Zhu Wang, Ruiping Li, Yan Liu, Wansen Sun, Xi Sun

**Affiliations:** ^1^ Department of Shaanxi Collaborative Innovation Center of Chinese Medicinal Resources Industrialization, Shaanxi University of Chinese Medicine, Xianyang, Shaanxi, China; ^2^ Department of Neurology, Second Affiliated Hospital, School of Medicine, Xi'an Jiaotong University, Xi'an, Shaanxi, China; ^3^ Clinical Laboratory, Shaanxi Provincial Hospital of traditional Chinese medicine, Xi'an, Shaanxi, China; ^4^ College of Pharmacy, Shaanxi University of Chinese Medicine, Xianyang, Shaanxi, China; ^5^ Department of Integrated Traditional Chinese and Western Medicine, Second Affiliated Hospital, School of Medicine, Xi’an Jiaotong University, Xi’an, Shaanxi, China; ^6^ Department of General Medicine, Second Affiliated Hospital, School of Medicine, Xi’an Jiaotong University, Xi'an, Shaanxi, China

**Keywords:** breast cancer, rs743572, polymorphism

## Abstract

Association between CYP17 T-34C (rs743572) polymorphism and breast cancer (BC) risk was controversial. In order to derive a more definitive conclusion, we performed this meta-analysis. We searched in the databases of PubMed, EMBASE and Cochrane for eligible publications. Pooled odds ratios (ORs) with 95% confidence intervals (95% CIs) were used to assess the strength of association between CYP17 T-34C polymorphism and breast cancer risk. Forty-nine studies involving 2,7104 cases and 3,4218 control subjects were included in this meta-analysis. In overall, no significant association between CYP17 T-34C polymorphism and breast cancer susceptibility was found among general populations. In the stratified analysis by ethnicity and source, significant associations were still not detected in all genetic models; besides, limiting the analysis to studies with controls in agreement with HWE, we also observed no association between CYP17 T-34C polymorphism and breast cancer risk. For premenopausal women, we didn't detect an association between rs743572 and breast cancer risk; however, among postmenopausal women, we observed that the association was statistically significant under the allele contrast genetic model (OR = 1.10, 95% CI = 1.03–1.17, *P* = 0.003), but not in other four models. In conclusion, rs743572 may increase breast cancer risk in postmenopausal individuals, but not in premenopausal folks and general populations.

## INTRODUCTION

Breast cancer (BC), the most frequent malignant neoplasm among female worldwide, accounts for approximate 25% of women malignant tumor. It is reported that 1.67 million people were diagnosed as BC ever year, therefore it has become a serious health issue, especially in the developing countries [1]. It is well known that the lifetime presence of the estrogen in the blood is an important pathogenic factor of BC, and this is in consistence with the low incidence of the breast cancer in males that is due to the lower estrogen levels and lower breast tissue volume. By now, researches on the status of hormone receptors and/or menopause associated with genetic alterations in BC risk have attracted an increasing number of attention, and lots of genes, including BRIPI, CHEK2, MDM, TGFB, TP53, BRCA1, BRCA2, and PTEN, and also several gene polymorphisms. Among genes of this family, CYP17, CYP19 and CYP1A1 have important functions in synthesis, metabolism and maintaining the levels of the androgen and estrogen hormones [2]. Previous published reasearches have demonstrated that estrogen act as a crucial role in the formation of BC; in addition, evidences have also been found about the positive role of cell surface receptors of estrogen in tumorigenesis [3]. Nevertheless, the precise mechanism behind estrogen in the formation of BC remains unknown. Previous studies have indicated that cytochrome P450c17α, which is a key enzyme in the synthesis of estrogen, and could increase the breast neoplasm risk [4]. The cytochrome P450c17α enzyme, predominantly catalyzes the formation of the precursor dehydroepiandrosterone (DHEA). Meanwhile, precursor DHEA could further be converted into estrogen through a succession of tissue-specific pathways [5, 6]. Estrogen, plays a vital part in the etiology of BC and identified the risk between estrogen and BC could well elucidate the biosynthesis and metabolism mechanisms. So far, more and more researches have demonstrated the correlation of estrogen-related genes genetic variations with BC risk. The CYP17T-34C (rs743572) polymorphism which is located on the human chromosome 10, in the 50-untranslated region has been most commonly reported [7].

Many studies about the genetic mutations or SNP occurring in CYP17 gene could enhance CYP17’s transcription rate and increase the enzyme cytochrome P450c17 level, resuling in an increasing number of bioavailable estrogen, which is likely to affect the risk and aggressiveness of BC [8]. But many previous article results between rs743572 mutations and BC risk remain conflicting: Han’s research [9] revealed that no statistically meaningful correlation of rs743572 with risk of BC. However, significant correlation was found between rs743572 and BC risk in another research on the same theme [10]. Since few new high-quality investigations were published, we performed this study to take a more precise evaluation of rs743572 with the risk of BC.

## RESULTS

### The main feature of included studies

**Figure 1:**
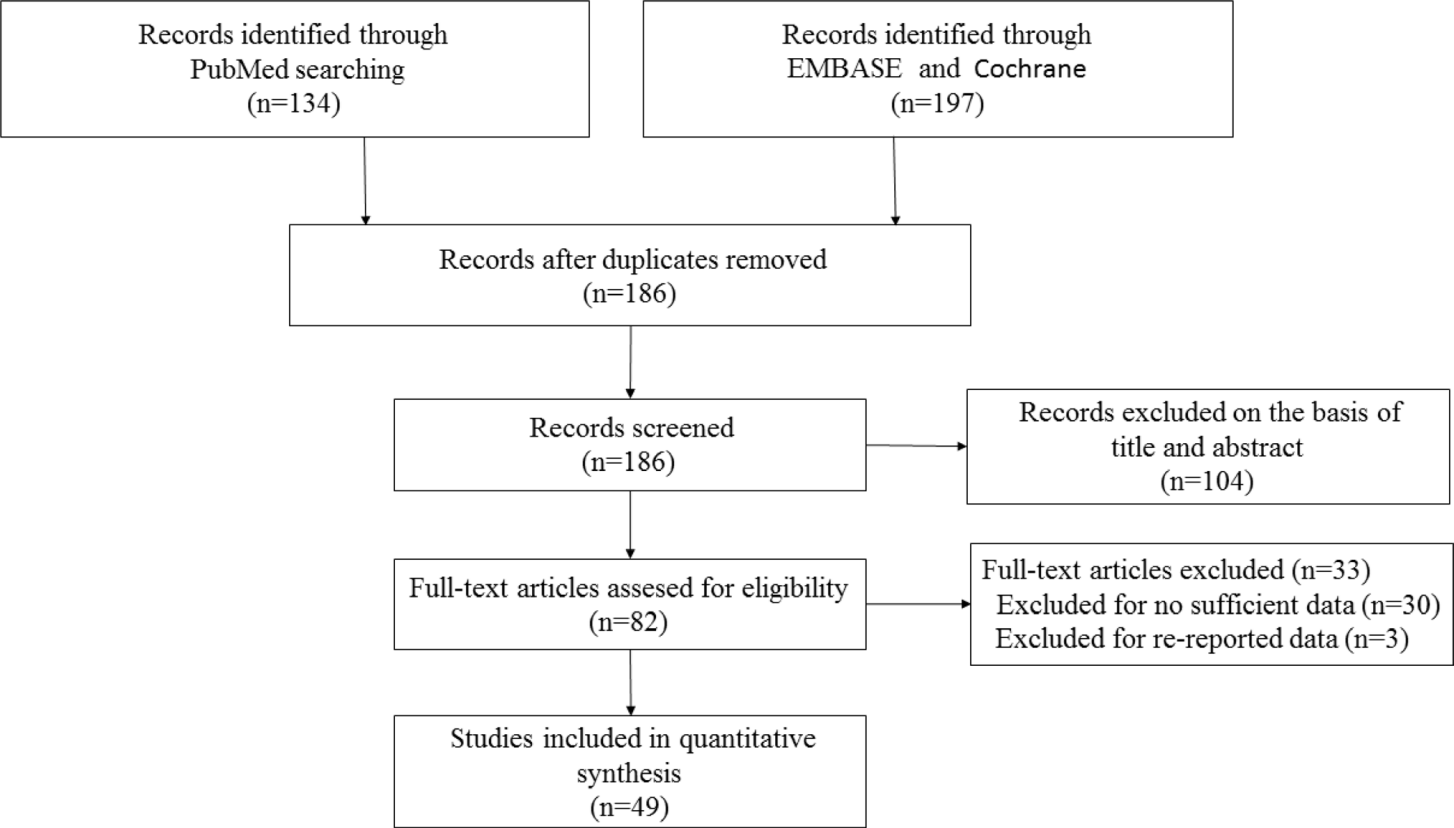
Flow diagram of the selection of the studies in this meta-analysis

As showed in Figure 1, 331 references were retrieved at first based on our selection strategy. 186 papers were remained after removing the duplicate reports. After reading titles and abstracts, we excluded 104 studies which were clearly unrelated. In the end, the whole of the rest of the papers were checked based on the inclusion and exclusion criteria. Finally, forty-nine studies on rs743572 and the risk of BC were eventually included in our study. Thirteen articles showed the number of three genotypes (TT, TC, and CC) among premenopausal women, and thirteen studies report TT, TC, and CC number in postmenopausal women. Main information of included studies were shown in Table 1. Among these qualified researches, seventeen were performed in Asians, twenty-five in Caucasians, one in Africans, one in both Asians and Caucasians, one in both Africans and Caucasians, and four in mixed ethnicity. Moreover, twenty-two studies were considered as moderate-quality studies (NOS scores of these researches were 4–6), and other twenty-seven studies were considered as high-quality studies (NOS scores of these studies were seven or above). Except for four included researches were not in agreement with Hardy–Weinberg equilibrium (HWE), genotype distributions in the control groups of other 45 researches were all satisfied with HWE.

**Table 1:** Characteristics of studies included in the meta-analysis

First author	Year	Country	Ethnicity	Source of control	Number (case/control)	HWE (*P* value)	NOS
Dunning [17]	1998	UK	Caucasian	PB	835/591	0.261	7
Weston [21]	1998	USA	Caucasian	HB	103/205	0.449	6
Weston [21]	1998	USA	African	HB	20/35	0.253	6
Helzlsouer [22]	1998	USA	Caucasian	PB	109/113	0.549	6
Bergman [23]	1999	Sweden	Caucasian	PB	109/117	0.304	6
Haiman [24]	1999	USA	Caucasian	PB	436/618	0.391	7
Huang [25]	1999	China	Asian	PB	123/126	0.972	6
Young [26]	1999	UK	Caucasian	PB	39/58	0.732	5
Kristensen [27]	1999	Norway	Caucasian	PB	510/201	0.351	7
Hamajima [28]	2000	Japan	Asian	HB	144/166	0.044	6
Kuligina [29]	2000	Russia	Caucasian	HB	240/182	0.017	6
Mitrunen [30]	2000	Finland	Caucasian	PB	479/480	0.967	7
Feigelson [18]	2001	USA	Mixed	PB	850/1508	0.335	7
Gudmundsdottir [31]	2003	Iceland	Caucasian	PB	500/395	0.131	7
Wu [32]	2003	Singapore	Asian	PB	188/671	0.512	6
Ambrosone [33]	2003	USA	Caucasian	PB	207/188	0.130	7
Tan [34]	2003	China	Asian	PB	250/250	0.117	7
Hefler [35]	2004	Austria	Asian	PB	388/1698	0.455	7
Ahsan [36]	2004	USA	Mixed	HB	313/271	0.457	6
Chacko [37]	2005	India	Asian	HB	140/140	0.133	6
Einarsdo´ttir [38]	2005	Sweden	Caucasian	PB	1499/1338	0.885	7
Shin [39]	2005	Korean	Asian	HB	462/337	0.134	7
Verla-Tebit [40]	2005	Germany	Caucasian	PB	527/904	0.380	7
Hopper [41]	2005	Australia	Caucasian	PB	1404/788	0.697	7
Onland-More [42]	2005	Netherlands	Caucasian	PB	335/373	0.189	7
Han [9]	2005	China	Asian	PB	210/427	0.037	6
Piller [43]	2006	Germany	Caucasian	PB	608/1298	0.062	7
Chakraborty [44]	2007	India	Asian	PB	186/212	0.550	6
Setiawan [45]	2007	USA	Mixed	PB	5147/6882	0.312	7
Chen [46]	2008	USA	Caucasian	PB	1037/1096	0.884	7
Sakoda [47]	2008	China	Asian	PB	615/877	0.232	7
Zhang [48]	2008	China	Asian	PB	299/342	0.454	7
Samson [49]	2009	India	Asian	PB	250/500	0.720	7
Sangrajrang [50]	2009	Thailand	Asian	HB	564/489	0.418	7
Sobczuk [51]	2009	Poland	Caucasian	PB	100/106	0.503	6
Antognelli [52]	2009	Italy	Caucasian	PB	547/544	0.982	7
Hosseini [53]	2009	Iran	Caucasian	HB	53/53	0.057	5
Jakubowska [54]	2009	Poland	Caucasian	HB	319/290	0.519	6
MARIE-GENICA [55]	2009	Germany	Caucasian	PB	3145/5487	0.254	7
Kato [56]	2009	USA	African	PB	184/189	0.152	6
Tuzuner [57]	2010	Turkey	Caucasian	PB	55/91	0.466	5
Syamala [58]	2010	India	Asian	HB	359/367	0.464	7
Surekha [59]	2010	India	Asian	PB	249/249	0.949	7
Iwasaki [60]	2010	Japan	Asian	HB	388/388	0.299	6
Iwasaki [60]	2010	Brazil	Asian	HB	78/79	0.144	6
Iwasaki [60]	2010	Brazil	Caucasian	HB	379/379	0.039	6
Kaufman [61]	2011	Mixed	Mixed	HB	1175/829	0.944	7
Cribb [10]	2011	Canada	Caucasian	HB	207/621	0.033	6
Ghisari [62]	2014	Inuit	Asian	PB	30/113	0.882	5
Chattopadhyay [63]	2014	India	Asian	PB	360/360	0.692	7
Karakus [64]	2015	Turkey	Caucasian	PB	199/197	0.934	6
Farzaneh [65]	2016	Iranian	Caucasian	PB	124/100	0.189	6

HWE: Hardy-Weinberg equilibrium for controls. PB: population-based study. HB: hospital-based study.

### Meta-analysis results

Meta-analysis results among overall populations, distribution of this polymorphism in case groups and control groups are presented in Table 2. For premenopausal women and postmenopausal women, distribution of this polymorphism in case groups and control groups are presented in Table 3, and the main outcome of our study are shown in Tables 4 and 5.

**Table 2:** Genotype distribution of the CYP17 (rs743572) polymorphism in cases and controls among overall populations

First author	Genotype (N)							
	Case				Control			
	Total	CC	CT	TT	Total	CC	CT	TT
Dunning	835	130	402	303	591	85	277	229
Weston	103	18	47	38	205	35	93	77
Weston	20	3	10	7	35	2	18	15
Helzlsouer	109	21	47	41	113	18	58	37
Bergman	109	15	62	32	117	9	55	53
Haiman	463	73	212	178	618	94	307	217
Huang	123	44	54	25	126	35	63	28
Young	39	5	13	21	58	7	28	23
Kristensen	510	67	241	202	201	26	101	74
Hamajima	144	20	83	41	166	27	95	44
Kuligina	240	47	111	82	182	44	77	61
Mitrunen	479	53	227	199	480	60	220	200
Feigelson	850	149	409	292	1508	227	739	542
Gudmundsdottir	500	60	247	193	395	66	173	156
Wu	188	69	82	37	671	229	333	109
Ambrosone	207	15	83	109	188	22	71	95
Tan	250	89	115	46	250	89	110	51
Hefler	388	75	186	127	1698	287	804	607
Ahsan	313	49	155	109	271	51	140	80
Chacko	140	6	40	94	140	3	22	115
Einarsdo´ttir	1499	238	711	550	1338	212	638	488
Shin	462	127	223	112	337	115	152	70
Verla-Tebit	527	103	244	180	904	157	424	323
Hopper	1404	230	621	553	788	113	364	311
Onland-More	335	44	140	151	373	50	157	166
Han	210	52	105	53	427	92	235	100
Piller	608	119	289	200	1298	236	596	466
Chakraborty	186	59	98	29	212	45	110	57
Setiawan	5147	833	2445	1869	6882	1070	3338	2474
Chen	1037	168	506	363	1096	175	523	398
Sakoda	615	216	297	102	877	298	441	138
Zhang	299	84	168	47	242	73	125	44
Samson	250	32	91	127	500	54	226	220
Sangrajrang	564	96	281	187	489	92	230	167
Sobczuk	100	46	44	10	106	34	55	17
Antognelli	547	60	258	229	544	68	249	227
Hosseini	53	6	29	18	53	13	33	7
Jakubowska	319	45	166	108	290	54	136	100
MARIE-GENICA	3145	529	1573	1043	5487	941	2712	1834
Kato	184	32	82	70	189	29	78	82
Tuzuner	55	10	27	18	91	9	44	38
Syamala	359	44	152	163	367	41	154	172
Surekha	249	9	69	171	249	16	95	138
Iwasaki	388	88	189	111	388	84	182	122
Iwasaki	78	13	48	17	79	23	33	23
Iwasaki	379	59	185	135	379	49	200	130
Kaufman	1175	171	581	423	829	124	392	313
Cribb	207	23	85	99	621	89	259	273
Ghisari	30	6	12	12	113	32	57	24
Chattopadhyay	360	14	116	230	360	7	93	260
Karakus	199	18	79	102	197	15	78	104
Farzaneh	124	22	70	32	100	17	56	27

**Table 3:** Genotype distribution of the CYP17 (rs743572) polymorphism in cases and controls among premenopausal women and postmenopausal women

First author	Genotype (*N*)							
	Case				Control			
	Total	CC	CT	TT	Total	CC	CT	TT
Helzlsouer	24	4	9	11	25	4	13	8
Bergman	109	15	62	32	117	9	55	53
Mitrunen	163	15	71	77	203	27	88	88
Wu	57	24	20	13	203	66	100	37
Ambrosone	96	7	31	58	86	10	28	48
Verla-Tebit	527	103	244	180	904	157	424	323
Chen	334	55	153	126	373	69	174	130
Samson	115	16	40	59	303	31	145	127
Antognelli	187	18	81	88	230	31	99	100
Kato	75	12	27	36	74	13	30	31
Zhang	150	38	87	25	124	37	67	20
Tan	95	32	45	18	97	30	40	27
Han	117	25	61	31	163	36	85	42
Helzlsouer	85	17	38	30	88	14	45	29
Mitrunen	316	38	156	122	277	33	132	112
Wu	131	45	62	24	468	163	233	72
Ambrosone	111	8	52	51	102	12	43	47
Einarsdo´ttir	1499	238	711	550	1338	212	638	488
Onland-More	335	44	140	151	373	50	157	166
Chen	680	111	339	230	677	96	333	248
Samson	134	16	50	68	197	23	99	75
Antognelli	360	42	177	141	314	37	150	127
Kato	109	20	55	34	115	16	48	51
Zhang	146	44	80	22	118	36	58	24
Tan	155	57	70	28	153	59	70	24
Han	93	27	44	22	264	56	150	58

**Table 4:** Meta-analysis results among overall populations

Comparisons	OR	95% CI	*P* (OR)	Heterogeneity		Effects model	*P* (Begg)	*P* (Egger)
				*I*^2^	*P*			
**Total**								
T VS C	0.99	0.96–1.01	0.281	37.1%	0.005	R	0.856	0.766
TT VS CC	0.99	0.98–1.01	0.309	18.6%	0.127	F	0.987	0.408
TC VS CC	0.98	0.93–1.03	0.365	0.80%	0.457	F	0.825	0.563
TT+TC VS CC	0.98	0.93–1.02	0.287	15.0%	0.182	F	0.975	0.574
TT VS TC+CC	0.99	0.95–1.02	0.463	30.5%	0.022	R	1.000	0.902
**Stratification by ethnicity**								
**Caucasian**								
T VS C	0.99	0.96–1.03	0.673	11.5%	0.294	F	-	-
TT VS CC	0.99	0.93–1.06	0.804	10.4%	0.233	F	-	-
TC VS CC	1.00	0.94–1.07	0.936	0.00%	0.467	F	-	-
TT+TC VS CC	1.00	0.94–1.06	0.604	11.1%	0.301	F	-	-
TT VS TC+CC	0.99	0.94–1.04	0.907	0.00%	0.596	F	-	-
**Asian**								
T VS C	0.97	0.89–1.06	0.574	60.8%	0.023	R	-	-
TT VS CC	0.99	0.95–1.02	0.483	33.9%	0.075	R	-	-
TC VS CC	0.97	0.88–1.07	0.525	11.4%	0.282	F	-	-
TT+TC VS CC	0.97	0.88–1.06	0.479	29.1%	0.114	F	-	-
TT VS TC+CC	0.97	0.84–1.11	0.652	60.0%	0.000	R	-	-
**African**								
T VS C	0.83	0.63–1.10	0.198	0.0%	0.621	F	-	-
TT VS CC	0.72	0.41–1.27	0.255	0.0%	0.393	F	-	-
TC VS CC	0.88	0.50–1.54	0.654	0.0%	0.363	F	-	-
TT+TC VS CC	0.80	0.47–1.36	0.408	0.0%	0.358	F	-	-
TT VS TC+CC	0.79	0.54–1.17	0.237	0.0%	0.859	F	-	-
**Stratification by Source**								
**PB**								
T VS C	0.98	0.95–1.00	0.102	35.2%	0.021	R	-	-
TT VS CC	0.95	0.90–1.01	0.073	18.7%	0.165	F	-	-
TC VS CC	0.95	0.90–1.00	0.047	0.0%	0.726	F	-	-
TT+TC VS CC	0.95	0.91–1.00	0.034	1.8%	0.439	F	-	-
TT VS TC+CC	0.99	0.95–1.02	0.474	32.4%	0.033	F	-	-
**HB**								
T VS C	1.03	0.97–1.09	0.299	38.9%	0.057	R	-	-
TT VS CC	1.10	0.97–1.24	0.128	18.2%	0.245	F	-	-
TC VS CC	1.14	1.02–1.28	0.024	0.0%	0.548	F	-	-
TT+TC VS CC	1.13	1.01–1.26	0.031	8.6%	0.355	F	-	-
TT VS TC+CC	0.99	0.91–1.08	0.847	30.6%	0.118	F	-	-
**Stratification by HWE**								
**Yes**								
T VS C	0.99	0.96–1.01	0.250	41.2%	0.002	R	-	-
TT VS CC	0.97	0.93–1.02	0.288	26.6%	0.050	R	-	-
TC VS CC	0.99	0.98–1.01	0.421	0.0%	0.501	F	-	-
TT+TC VS CC	0.99	0.99–1.02	0.264	15.6%	0.180	F	-	-
TT VS TC+CC	0.98	0.95–1.02	0.381	35.3%	0.010	R	-	-

F: fixed effects model; R: random effects model.

**Table 5:** Meta-analysis results among premenopausal women and postmenopausal women

Comparisons	OR	95% CI	*P* (OR)	Heterogeneity		Effects model	*P* (Begg)	*P* (Egger)
				*I*^2^	*P*			
**Premenopausal**								
T VS C	1.02	0.93–1.10	0.717	17.6%	0.267	F	-	-
TT VS CC	1.01	0.85–1.20	0.885	6.4%	0.383	F	-	-
TC VS CC	0.97	0.83–1.14	0.709	0.0%	0.492	F	-	-
TT+TC VS CC	1.04	0.92–1.18	0.513	21.4%	0.227	F	-	-
TT VS TC+CC	0.95	0.81–1.10	0.467	29.3%	0.151	F	-	-
**Postmenopausal**								
T VS C	1.10	1.03–1.17	0.003	10.6%	0.339	F	-	-
TT VS CC	0.96	0.84–1.10	0.539	0.0%	0.835	F	-	-
TC VS CC	0.96	0.85–1.08	0.478	0.0%	0.902	F	-	-
TT+TC VS CC	0.96	0.85–1.08	0.467	0.0%	0.930	F	-	-
TT VS TC+CC	0.99	0.90–1.08	0.796	8.9%	0.357	F	-	-

F: fixed effects model; R: random effects model.

**Figure 2:**
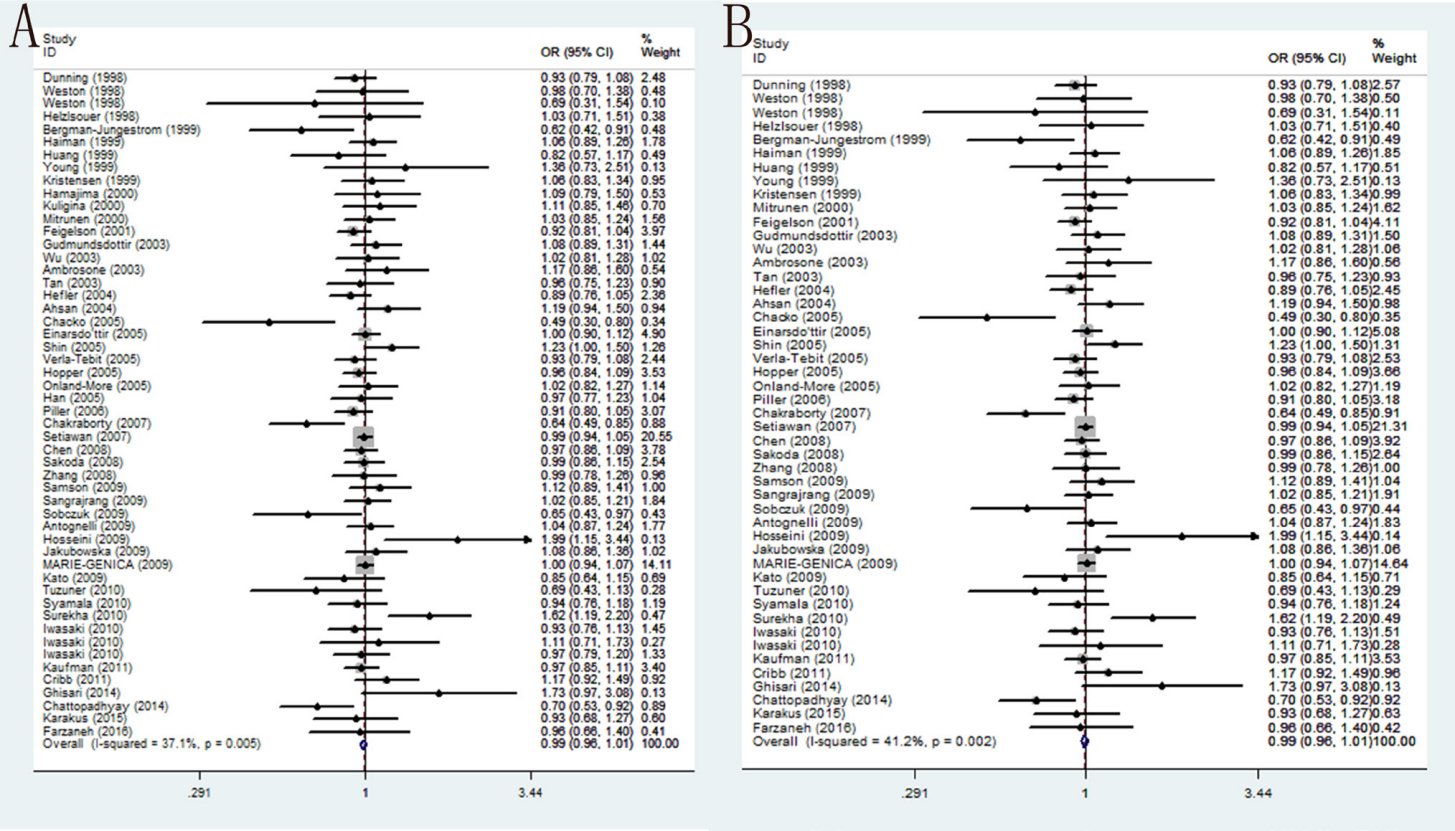
Forest plots of associations between rs743572 and breast cancer risk (**A**) the overall populations in the allele contrast genetic model; (**B**) limiting the analysis to studies with controls in agreement with HWE under the allele contrast genetic model.

In overall populations, the association of CYP17 T-34C polymorphism with BC susceptibility was studied in forty-nine researches including 27,104 cases and 34,218 controls. No significant correlation was found between this polymorphism and BC susceptibility among any of the five genetic models: T/C (OR = 0.99, 95% CI = 0.96–1.01, *P* = 0.281), TT/CC (OR = 0.99, 95% CI = 0.98–1.01, *P* = 0.309), TC/CC (OR = 0.98, 95% CI = 0.93–1.03, *P* = 0.365), TT+TC/CC (OR = 0.98, 95% CI = 0.93–1.02, *P* = 0.287) and TT/TC+CC (OR = 0.99, 95% CI = 0.95–1.02, *P* = 0.463). Analogously, further subgroup analysis by ethnicity and source found similar results that in all the ethnic groups, HB group and PB group there is no significant correlation between rs743572 and BC susceptibility. Moreover, if we only analyze the studies with controls in agreement with HWE, no correlation between rs743572 and BC risk were observed (Table 4) (Figure 2).

**Figure 3:**
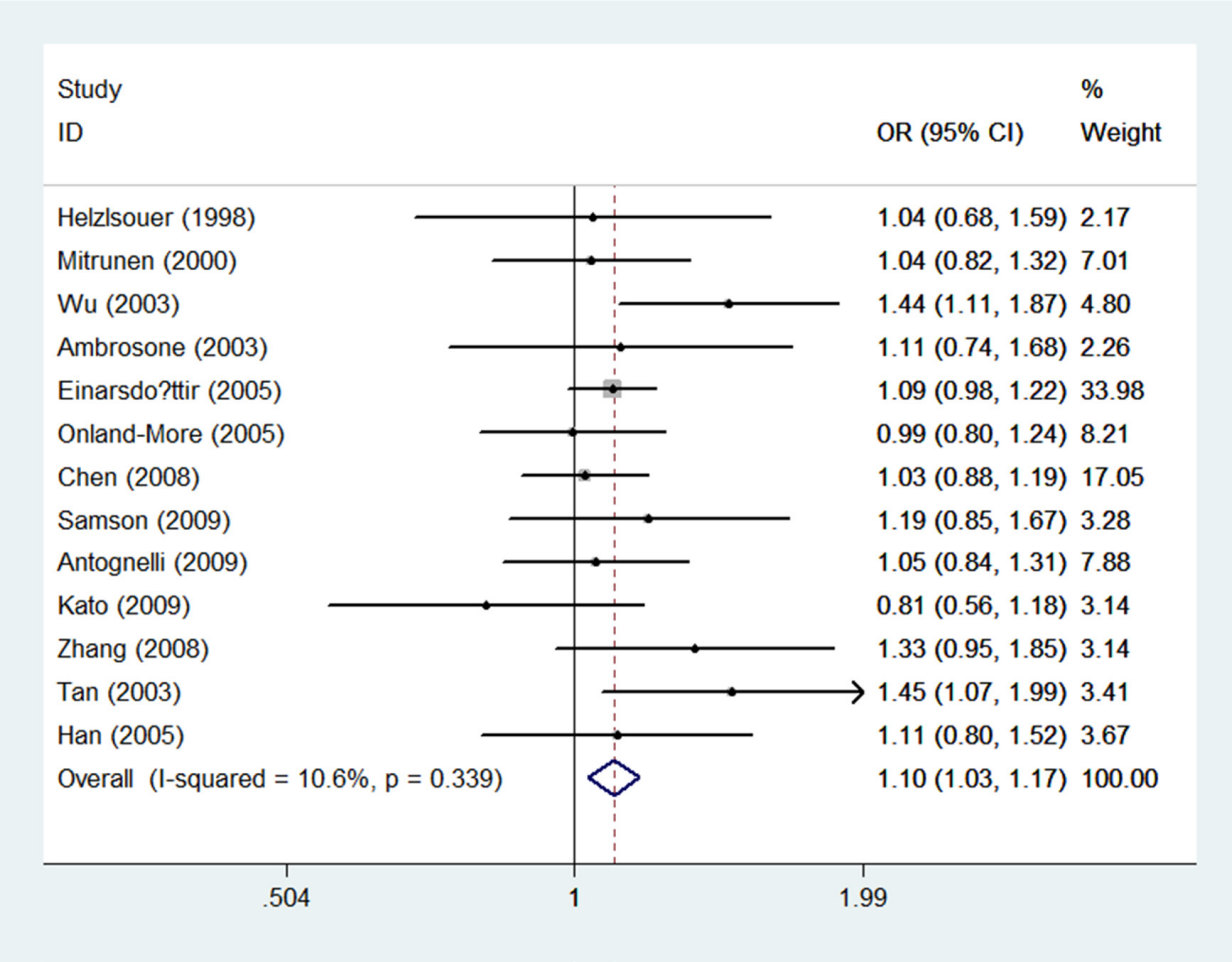
Forest plots of associations between rs743572 and breast cancer risk among postmenopausal women in the allele contrast genetic model

In premenopausal individuals, thirteen included researches with 2, 029 breast cancer case groups and 2, 920 control groups were eventually included. There is no statistical correlation of rs743572 with breast cancer susceptibility in T/C model, the TT/CC, the TC/CC, the TT+TC/CC, and the TT/TC+CC (OR = 1.02 with 95% CI 0.93–1.10, OR = 1.01 with 95% CI 0.85–1.20, OR = 0.97 with 95% CI 0.83–1.14, OR = 0.95 with 95% CI 0.81–1.10, and OR = 1.04 with 95% CI 0.92–1.18, respectively). In postmenopausal women, significant correlation was found in T/C model (OR = 1.10, 95% CI = 1.03–1.17, *P* = 0.003) (Table 5) (Figure 3). However, there were no significant associations between the rs743572 polymorphism and breast cancer risk in other genotype distributions: TT/CC (OR = 0.96, 95% CI =0.84–1.10, *P* = 0.539), TC/CC (OR = 0.96, 95% CI =0.85–1.08, *P* = 0.478), TT+TC/CC (OR = 0.96, 95% CI =0.85–1.08, *P* = 0.930) and TT/TC+CC (OR = 0.99, 95% CI =0.90–1.08, *P* = 0.357) (Table 5).

### Sensitivity analysis

Even though four researches included in our studies were not conformed to the HWE balance (*P* < 0.05), final consequences were not changed when we excluded the abovementioned four studies. Besides, after performing the sensitivity analysis, the pooled OR values were not statistically significant changed when we delete each of the researches, indicating that this study has good stability and reliability.

### Heterogeneity analysis

Heterogeneity was obtained by Q statistic. When the *P* value more than 0.1 in the *Q* test, then the fixed-effect models were selected to conduct relevant statistical analysis; otherwise, random-effect models were selected.

### Publication bias

**Figure 4:**
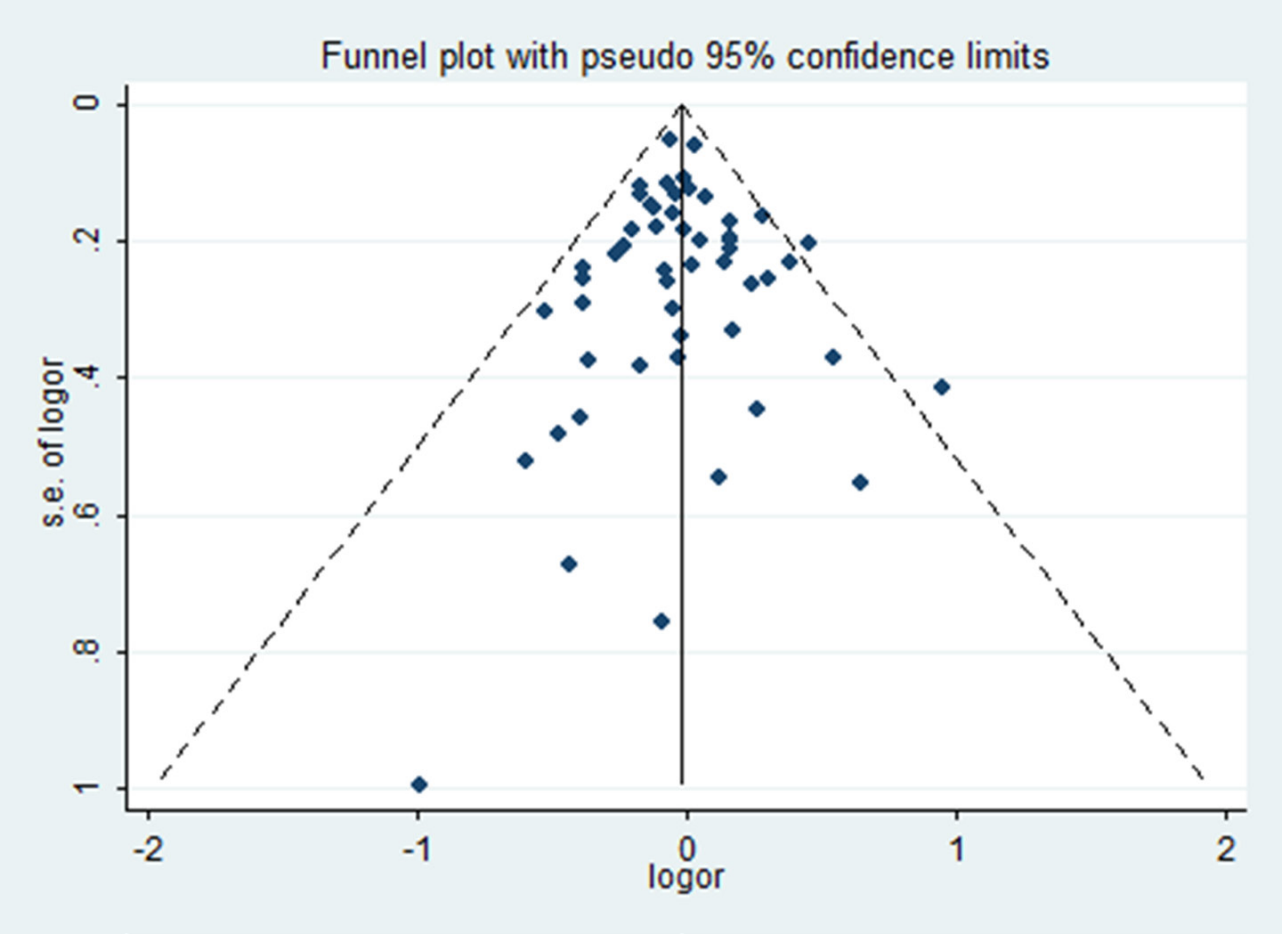
Funnel plots of rs743572 and breast cancer risk in the heterozygote genetic model

No statistical evidence of publication bias was found in the Begg’s test and Egger’s test. What’s more, funnel plot also did not show any evidence of obvious asymmetry (Table 4) (Figure 4).

## DISCUSSION

With the popularization and the rapid development of technology in the field of medicine, people have a deeper recognition of breast cancer. However, the specific mechanisms of the occurrence and the development of this cancer remain unclear. It is well established that estrogen involves in the development of mammary gland and plays crucial role in initiating of BC [2]. Extensive evidences have also been demonstrated that lifetime exposure to endogenous and/or exogenous estrogen, increased the risk of the morbidity of breast cancer [11]. Besides, estrogen plays a positive role of cell surface receptors of in tumorigenesis [2, 3]. Significance of genes functioning in steroid hormone synthesis is well established in breast cancer susceptibility. CYP17, a commonly known gene could code for the cytochrome P450c17α enzyme that is one of the key enzymes participated in estrogen biosynthesis [4]. CYP17 T-34C polymorphism, in the region (5′-UTR) of CYP17, has been reported up-regulate CYP17 transcription in some studies but not in others [7]. The functional impact of the T/C change is still an unresolved mystery. Moreover several studies have reported conflicting results with respect to menopausal status and CYP17 polymorphism. Hence, for the purpose of acquire a more accurate assessment of the association between rs743572 and BC risk we performed this meta-analysis whose included research studies identified in the PubMed, EMBASE and the Cochrane.

In overall populations, our results indicate no significant correlation between rs743572 and the risk of BC. Similar results could be obtained when stratified by ethnicity in all ethnic groups. In addition, confining the analysis to the researches with control groups in consistent with HWE, we also observed no correlation between rs743572 and risk of BC. Nevertheless, meaningful correlation was showed between rs743572 and breast neoplasm risk in Russian individuals [10]. There were three meta-analyses, all published in 2010, including 24–43 papers from different populations and demonstrated no association between the rs743572 and BC, which further demonstrate that our results are credible [12, 13, 14].

Estrogen is mainly produced in the ovaries and mammary glands among premenopausal women. However, in postmenopausal individuals, adipose tissue mainly acts as an important part in estrogen biosynthesis [15, 16]. Several studies have reported conflicting results of menopausal and CYP17 polymorphism: the study by Dunning *et al.* [17] showed the association between increased A2 genotype and premenopausal breast cancer; while Feigelson *et al.* [18] reported increasing frequency of A2 genotype associated with postmenopausal BC patients. We observed that rs743572 was correlated with an increasing BC risk among postmenopausal women under the allele contrast genetic model, but not in other models; however, no association was found in premenopausal women. Previous published meta- analysis reported that no association existed both in postmenopausal women and among premenopausal women [12, 13, 14]. Compared with them, our study used five genetic models to reduce the probability of class I errors, so our result was more reliable.

Unavoidable, there are some limitations in meta-analysis. First, breast cancer is a multifactorial disease involving genetic and environmental interactions; however, it was still not addressed the impact of gene–environmental interactions in this meta-analysis [19]. Second, the detailed individual information in some studies was unknown; thus, we could not assess the susceptibility of breast cancer according to other risk factors including obesity, family history, radiation therapy in young age, history of pregnancy, breast-feeding, hormone therapy and so on [20]. Last, there are only two studies about Africans, more well designed studies with different population should be performed to make more persuasive conclusions. In summary, our results indicate that rs743572 could increase risk of BC in postmenopausal individuals, but not in premenopausal women and the general population. Further multicenter research with complete risk factors are required to validate the potential role of rs743572 polymorphism in BC. More multicenter studies and complete risk factors are needed to further confirm the possible role of rs743572 polymorphism in the occurrence and development of breast cancer.

## MATERIALS AND METHODS

### Literature and search strategy

We searched the PubMed, EMBASE and Cochrane databases for studies performed prior to March 7, 2017 that reported an association between rs743572 SNP and breast cancer risk. There were no language restrictions in our searching process. The searching strategy was as follow: (breast cancer OR breast carcinoma) AND (polymorphism OR variant OR genotype OR SNP) AND (CYP17 OR CYP17A1 OR P450c17). Besides, the references of the retrieved studies were also reviewed to identify additional eligible studies.

### Inclusion criteria

The included studies must meet the following criteria: (1) case-control design; (2) investigating the association between CYP17 T-34C polymorphism and breast cancer risk; (3) sufficient genotyping data that could be used to calculate odds ratios (ORs) and 95% confidence intervals (CIs); (4) all the breast cancer subjects in case groups must be pathologically confirmed. The exclusion criteria were: (1) not case-control studies; (2) review article or commentary; (3) duplicate studies; (4) studies lacking relevant data.

### Data extraction

Two reviewers independently extracted the relevant data from the included studies, and discrepancies were resolved during a discussion with a third author. The following information was extracted: the first author, year of publication, country, ethnicity, source of controls, number of cases and controls, and *P* value for Hardy-Weinberg equilibrium (HWE). In addition, we also evaluated the methodological quality of included studies based on Newcastle-Ottawa Scale (NOS), which scored studies according to three aspects: selection, comparability, and exposure. Therefore, all studies could be divided into three categories: “low quality” studies (score 0–3); “moderate quality” studies (score 4–6); “high quality” studies (score 7–9).

### Statistical analysis

The association between CYP17 T-34C polymorphism and BC susceptibility was measured by pooled odds ratios (ORs) and 95% confidence intervals (CIs) in five genetic models, including an allele contrast genetic model, a homozygote genetic model, a heterozygote genetic model, a dominant genetic model, and a recessive genetic model. Pooled ORs were performed for homozygote comparison (TT vs. CC for rs743572), heterozygote comparison (TC vs. CC for rs743572), dominant model (TT/TC vs. CC for rs743572), recessive model (TT vs. TC/CC for rs743572) and allelic model (T vs. C for rs743572) respectively. Statistical heterogeneity was evaluated by *I*^2^ test and *Q* test, *P* < 0.05 was considered statistically significant. For *I*^2^ test, the criteria for heterogeneity were as follows: *I*^2^ < 25%, no heterogeneity; 25%–75%, moderate heterogeneity; *I*^2^ > 75%, high heterogeneity. If the *P* value of *Q* test was < 0.1, the random-effects model was used; otherwise, the fixed-effects model was applied. Sensitivity analysis was performed by excluding one study at a time to assess the influence of each study on the pooled ORs. Begg’s funnel plot and Egger’s tests were used to examine publication bias and to evaluate the stability of the results by sensitivity analysis. The *P* value for Hardy-Weinberg equilibrium (HWE) in controls of every included study was calculated by Chi-square test. Subgroup analysis was performed according to ethnicity. All statistical analyses were performed using STATA version 10.0 software (StataCorp LP, College Station, TX, USA). All *P* values were two sided, and *P* < 0.05 was considered statistically significant.
